# Erythroderma: A Rare Complication of Dyshidrotic Eczema

**DOI:** 10.7759/cureus.3005

**Published:** 2018-07-19

**Authors:** Osman Bhatty, Leah Grant, Jian Shen, Agnes Colanta, Scott Lauer, Christopher Huerter

**Affiliations:** 1 Department of Internal Medicine, Creighton University, Omaha, USA; 2 Creighton University School of Medicine, Department of Internal Medicine, Omaha, USA; 3 Department of Pathology, Creighton University School of Medicine, Omaha, USA; 4 Department of Pathology, University of Nebraska Medical Center, Omaha, USA; 5 Department of Dermatology, Creighton University School of Medicine, Omaha, USA

**Keywords:** erythroderma, eczema, dyshidrotic

## Abstract

A 47-year-old man with a history of dyshidrotic eczema presented to the emergency department with diffuse erythema, chills and pruritus of three weeks’ duration. The patient had received two injections of methotrexate in the preceding two weeks, both of which had failed to improve his whole-body erythema and pruritus. In the emergency department, the patient was evaluated for infection and admitted for the dermatology consultation. After being seen on the general medical floor by the dermatology service the diagnosis of erythroderma was made and the patient was treated with intravenous (IV) cyclosporine therapy, with which his rash dramatically improved over three days. This case report summarizes the presentation and differential of erythroderma, and highlights the importance of having a high index of suspicion for this potentially fatal disease.

## Introduction

Erythroderma is an uncommon disorder that has a broad differential and requires a high index of suspicion to treat. Its varied underlying etiologies require significantly different approaches to treatment.

## Case presentation

**Figure 1:**
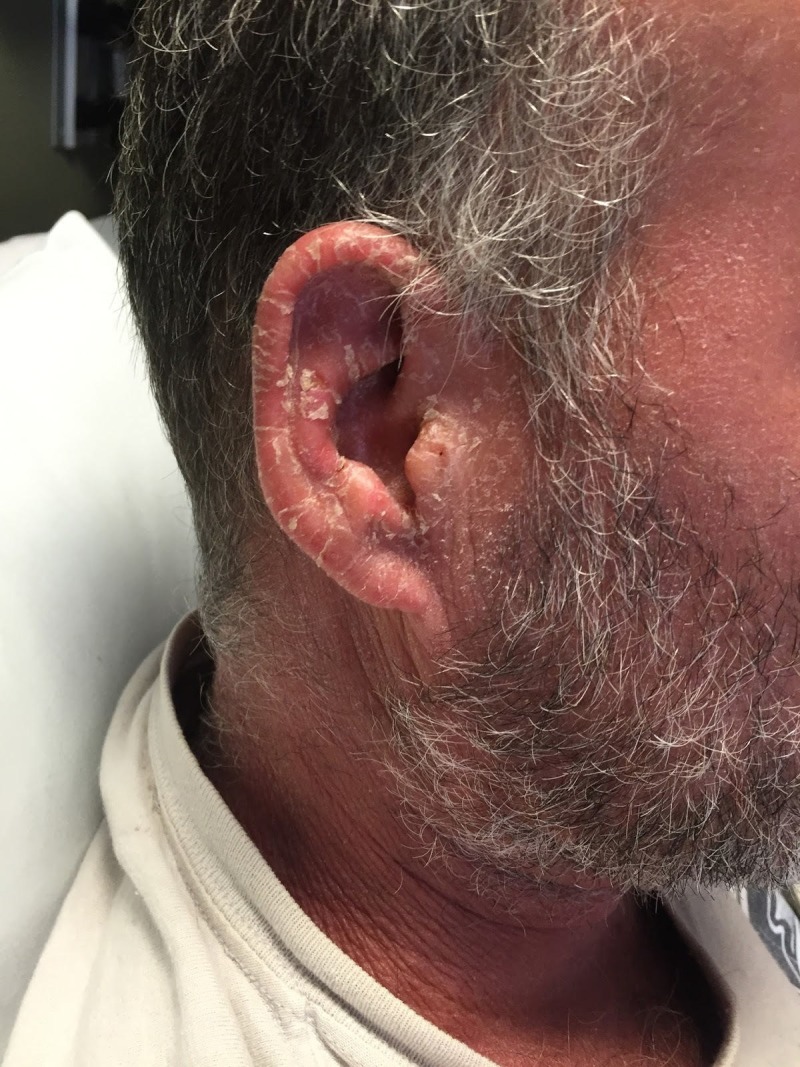
Diffuse erythema.

**Figure 2:**
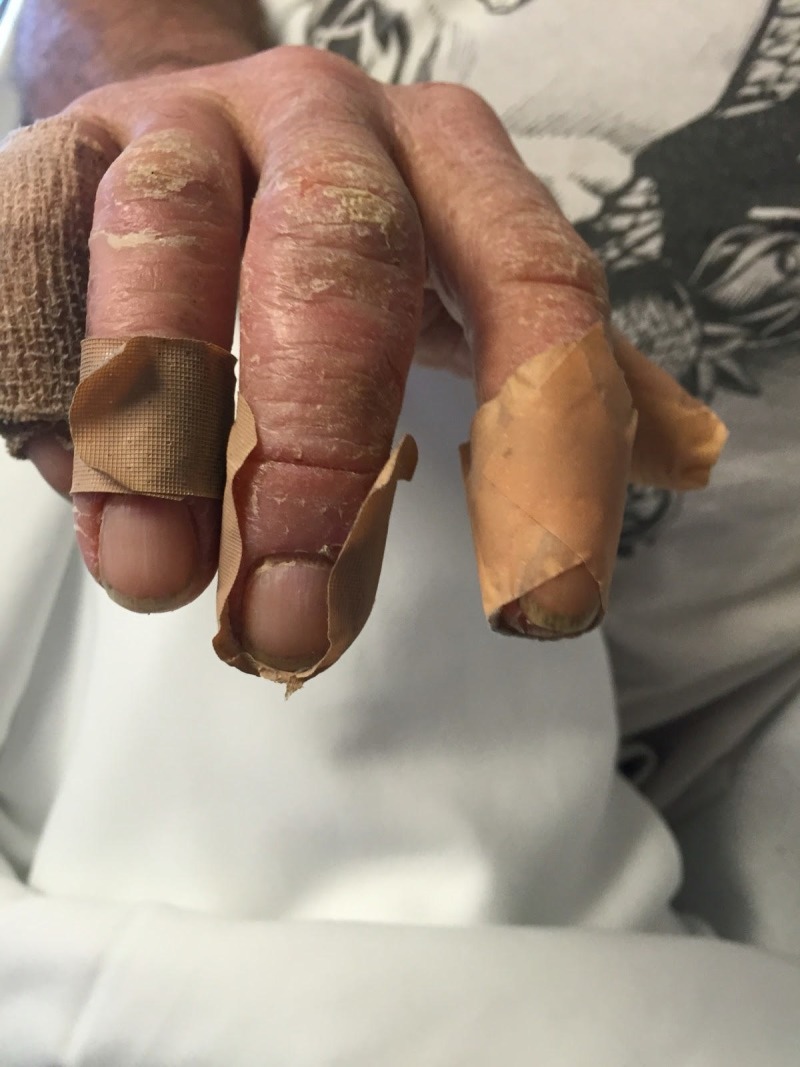
Scaling patches and plaques of hand.

**Figure 3:**
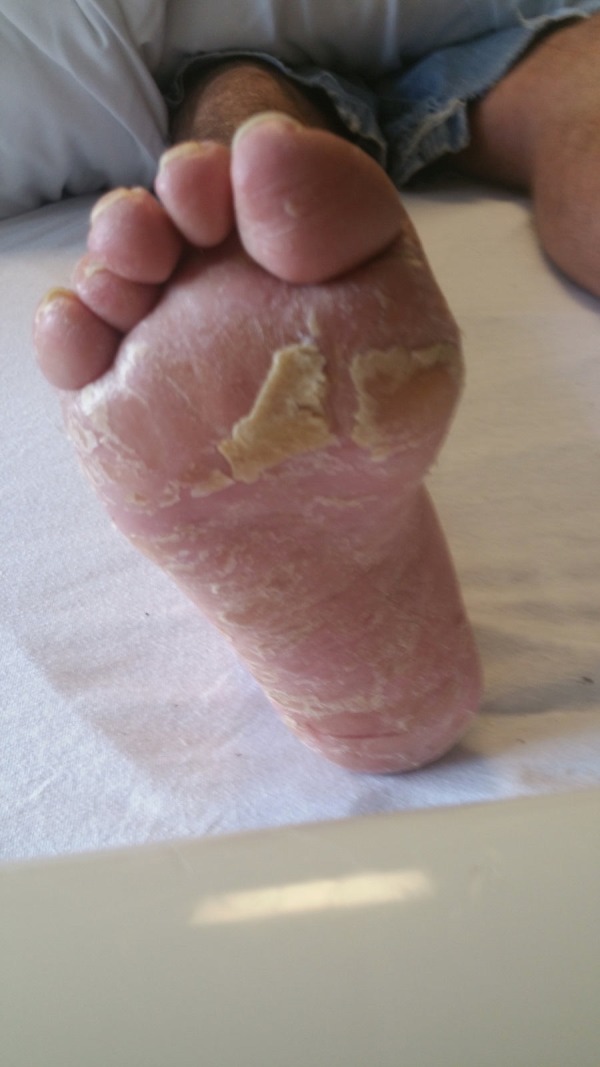
Exfoliation and inflammation of right foot.

**Figure 4:**
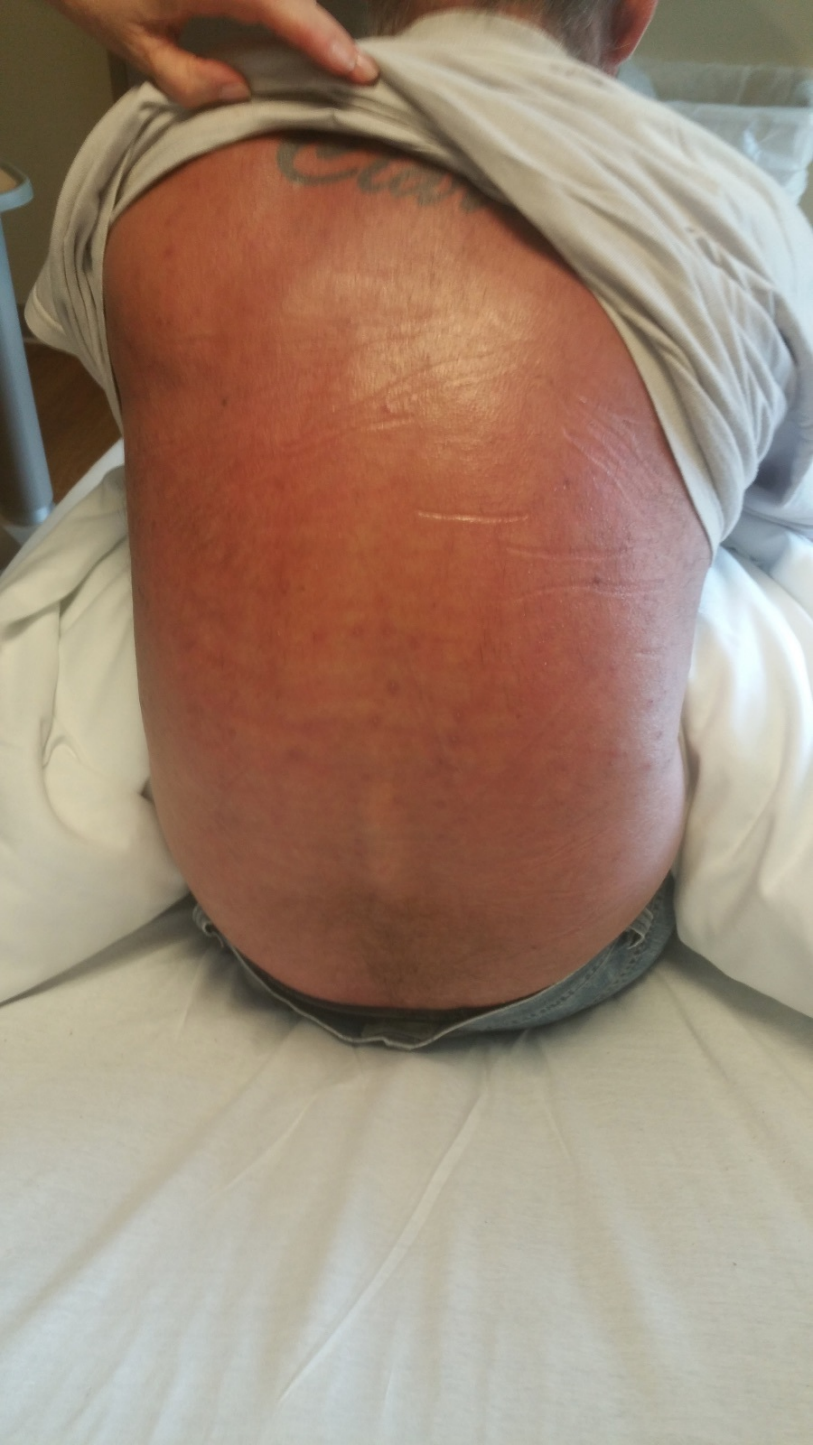
"Red man's syndrome" – diffuse erythema.

A 47-year-old Caucasian male presented to the emergency department (ED) for diffuse erythema and pruritus of three weeks’ duration. The patient had been followed in dermatology clinic for hand-foot dyshidrotic eczema for approximately three years prior to his admission. The patient’s eczema had been well controlled on low dose weekly oral methotrexate for two years until he reported no longer being able to tolerate the pills. At that time oral methotrexate was discontinued and topical betamethasone was begun. Three weeks prior to admission the patient developed whole body erythema with pruritus. He had been seen in the dermatology clinic twice and received two injections of methotrexate every seven days. He presented to the ED one week after the second injection having failed therapy with a worsening diffuse rash. At the time of presentation to the ED, the patient had diffuse erythema covering >90% of his body (Figures 1-4), generalized exfoliation, and was visibly shaking with chills. Blood cultures and human immunodeficiency virus (HIV) testing were negative. Electrocardiogram (EKG), chest X-ray (CXR), comprehensive blood count (CBC), comprehensive metabolic panel (CMP) and urine analysis (UA) were within normal limits. With skin biopsy results pending, the patient was started on 5 mg/kg/day of intravenous (IV) cyclosporine along with topical triamcinolone cream applied twice a day (BID). Over the course of three days, the patient improved dramatically. A punch biopsy of skin was diagnosed as spongiotic dermatitis. The differential diagnosis included drug eruption, contact hypersensitivity reaction, and other eczematous dermatitis.

A punch biopsy of skin showed hyperkeratosis, parakeratosis, mild irregular acanthosis, and mild spongiosis. The granular layer was maintained, and rare Langerhans cell microabscesses were seen in the epidermis. Spongiosis involving the follicular epithelium was identified. Within the dermis, there was a superficial perivascular mixed inflammatory infiltrate of lymphocytes and scattered eosinophils. These histopathological features are most consistent with spongiotic dermatitis. Spongiotic dermatitis is a reaction pattern that is often seen in patients with erythroderma and is relatively nonspecific for an underlying etiology. Given the patient’s history, an acute exacerbation of the patient’s dyshidrotic eczema with secondary autoeczema progressing to erythroderma was favored. The histologic differential diagnosis also included a drug reaction or contact hypersensitivity reaction. Psoriatic erythroderma often has diminution or loss of the granular cell layer and intraepidermal neutrophils, which were not present in the current biopsy. Pityriasis rubra pilaris may show some epidermal spongiosis but in a classic case will show mild psoriasiform epidermal hyperplasia, follicular plugging, and alternating orthokeratosis and hyperkeratosis in the cornified layer, both horizontally and vertically (so-called “checkerboard pattern”), findings which were absent in this case. The absence of an atypical epidermotropic lymphoid infiltrate argued against the possibility of mycosis fungoides (cutaneous T-cell lymphoma).

**Figure 5:**
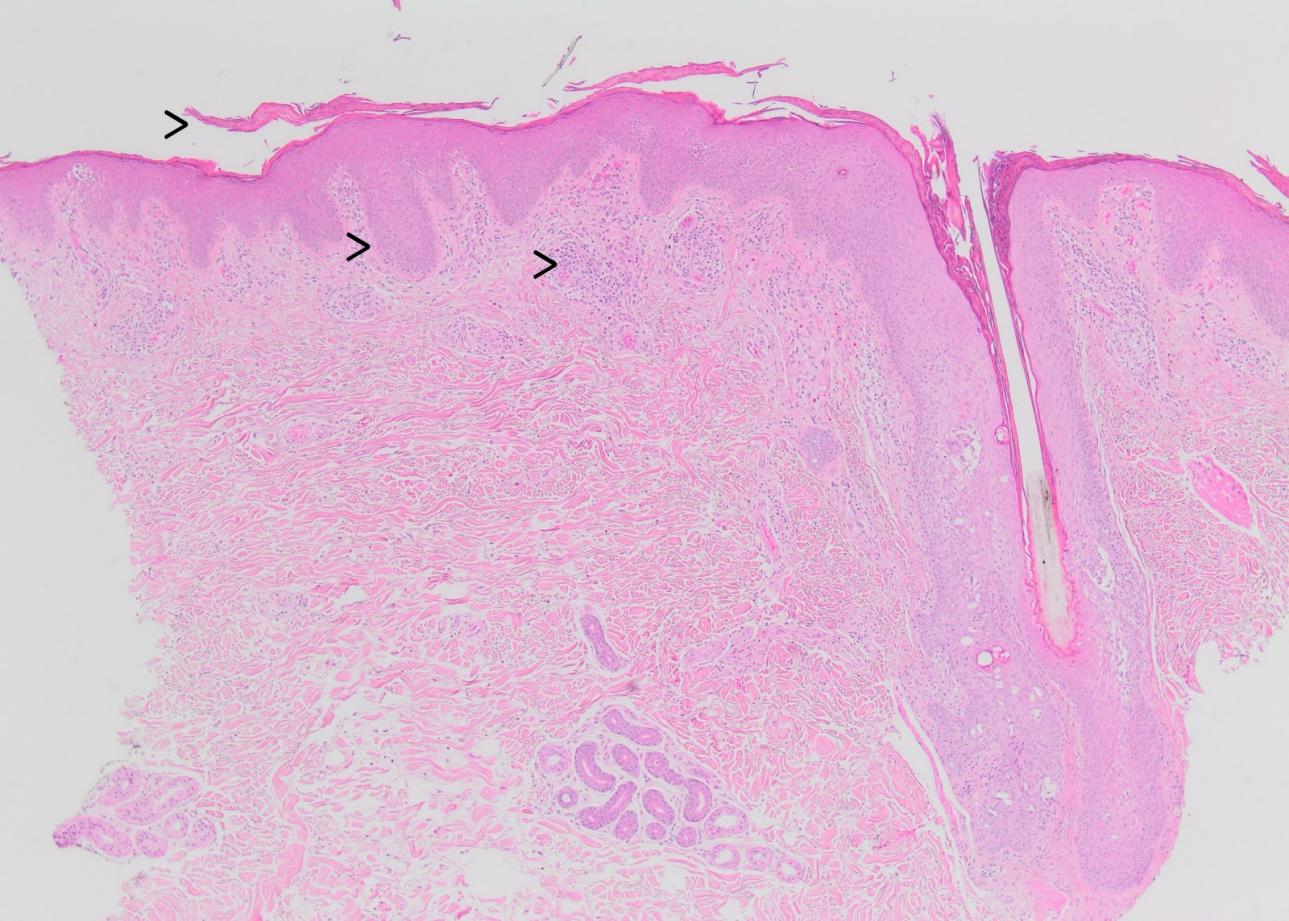
(Left to right) The skin biopsy shows parakeratosis with irregular acanthosis and superficial perivascular mixed inflammatory. In the field, an intact hair follicle is also seen (H&E stain X 40).

**Figure 6:**
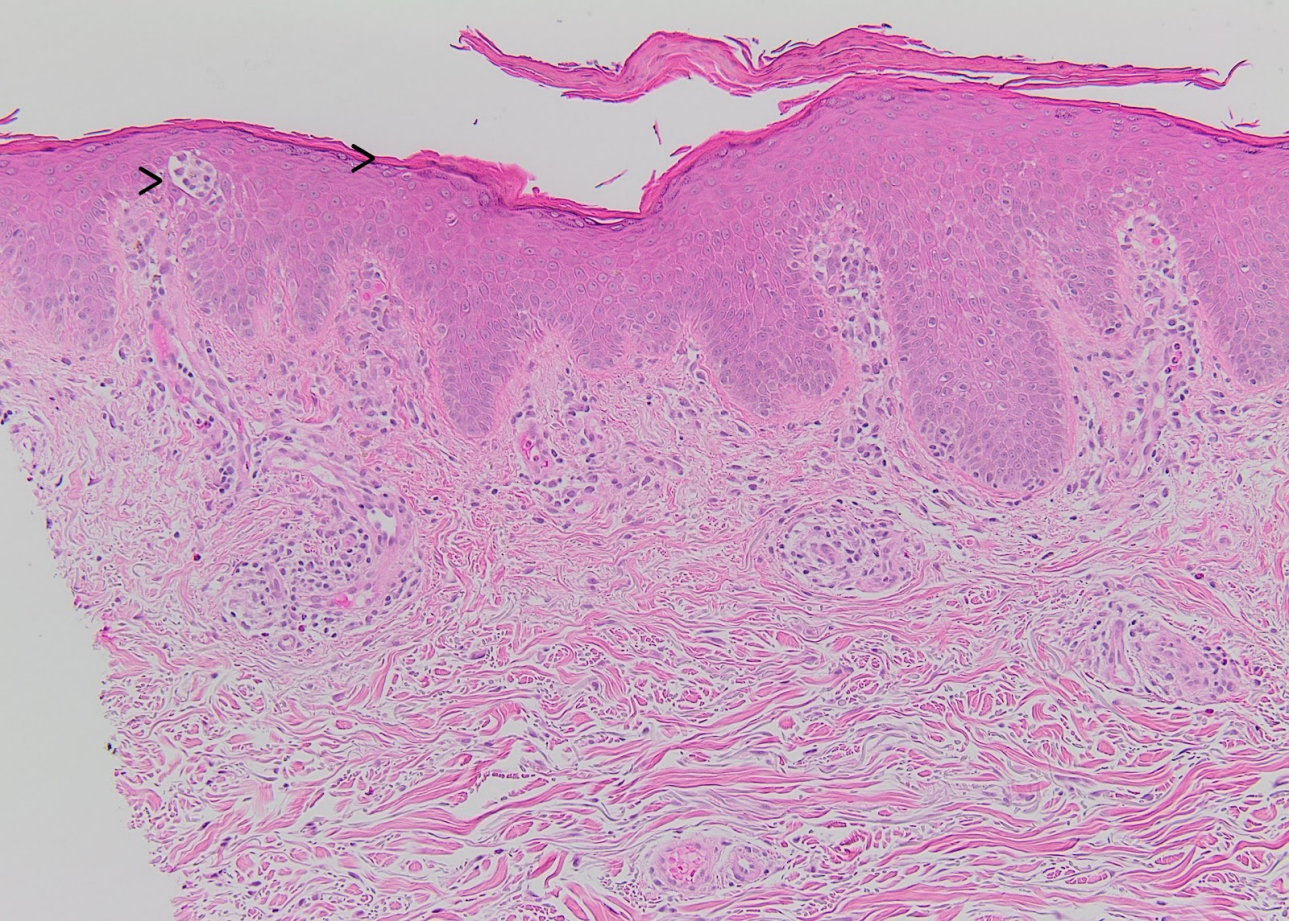
(Left to right) In the epidermis, rare Langerhans cell microabscesses while the granular layer is maintained (H&E stain X 100).

The skin biopsy shows parakeratosis (Figure 5) with irregular acanthosis and superficial perivascular chronic inflammation. In the lower right aspect of the field, there is a hair follicle with spongiosis involving the follicular epithelium (haematoxylin and eosin (H&E) stain X 40). In the epidermis (Figure 6), the granular layer is maintained and rare Langerhans cell microabscesses are identified (H&E stain X 100). The epidermis shows diffuse mild spongiosis. In the dermis, superficial perivascular lymphohistiocytic infiltrate with admixed eosinophils. Atypical lymphocytes with epidermotropism were not present.

The patient followed up approximately a month and a half to dermatology clinic during which time he had clinically improved on his low dose cyclosporine (100 mg twice a day). He continued this dose for a total of four months until his rash had almost completely resolved after which he was instructed to use clobetasol ointment as needed.

## Discussion

Erythroderma is a generalized exfoliative dermatitis of the skin that was first described by Ferdinand Ritter von Hebra in 1868. It is an inflammatory disorder with many underlying etiologies that may give rise to systemic complications which can become life-threatening. Quick recognition of this disease is paramount to the safety of the patient [1].

Overall epidemiologic studies of erythroderma have shown a male predominance that ranges from 2:1 to 4:1 [2] and an average reported age of 41 to 61 years when excluding children [1]. The true global incidence of erythroderma is unknown – large prospective studies in the Indian subcontinent have shown incidence to be 35 per 100,000 [3]. Studies in the Netherlands have shown an incidence of .9 to 100,000 [3,4]. It is considered a rare disease with few published studies concerning epidemiology [5].

Etiology is certainly of interest with erythroderma because the differential is extensive. The most common cause has been shown to be an exacerbation of preexisting dermatoses [3,6] as in the case of our patient, but psoriasis, spongiotic dermatitis, drug reactions and cutaneous T-cell lymphomas are common causes as well [3,7]. Patients with erythroderma secondary to psoriasis usually have a history of localized disease before onset. Triggers include withdrawal of steroids, discontinuation of methotrexate (our patient had a history of stopping the medication due to intolerance), topical irritants, systemic medications such as antimalarials, lithium, terbinafine, infections, emotional stress and other illnesses. Presentation may have the classic plaques of psoriasis and can be evident early along with characteristic nail changes [2]. Erythroderma due to drug changes has a rapid onset particularly if the patient had prior exposure to a medicine and developed a hypersensitivity [8]. Hypersensitivity may develop 2-5 weeks after starting the agent [2]. Pityriasis rubra pilaris begins as a seborrheic dermatitis-like presentation of the scalp and becomes a generalized salmon-colored erythema after sun exposure that is more classic of erythroderma. Follicular pink papules affecting the dorsal fingers, wrists and elbows can be present along with “islands of spared skin” on the torso [2].

In a recent etiological study of 103 patients with erythroderma, the most common clinical symptoms included some degree of scaling, pruritus, diffuse erythema and fever. Peripheral pitting edema was found in almost half of the patients along with palmar/plantar hyperkeratosis and nail changes [5]. Slightly less than half of the patients had splenomegaly and hepatomegaly, and slightly less than half had lymphadenopathy. The most common laboratory findings were increased inflammatory markers (erythrocyte sedimentation rate and C-reactive protein) (96.1%), decreased hemoglobin (30.1%), and leukocytosis (48.5%). Skin biopsy was done in 92.2% of patients which lead to a diagnosis in two-thirds of cases [5]. Histological analysis usually demonstrates an epidermal perivascular infiltrate of lymphocytes and eosinophils, dilated capillaries and hyperkeratosis [7].

The pathogenesis of erythroderma is currently unclear but likely involves adhesion molecules and ligands which play a significant role in the infiltration of lymphocytes and mononuclear cells during inflammation [3]. The increase in adhesion molecule expression stimulates dermal inflammation which leads to the proliferation of epidermal cells and an increase in inflammatory mediators [3,9]. Further understanding of these pathways may lead to more targeted therapies in the future.

Finally, systemic complications of erythroderma include cutaneous infection, sepsis, fluid and electrolyte dysregulation, thermoregulatory problems and high output cardiac failure due to shunting large volumes of blood to the skin [3,9]. These serious complications underscore the need for high clinical suspicion and treatment. Inflamed skin can become an open portal for bacteria, increasing the likelihood for cellulitis and bacteremia. Erythroderma can increase protein loss by 25-30% in psoriatic type which can cause a negative nitrogen balance manifesting as muscle wasting, edema and hypoalbuminemia [2].

Treatment is highly dependent on underlying etiology but in cases where the underlying cause remains elusive empiric treatment can be considered with agents such as systemic corticosteroids, methotrexate, cyclosporine or mycophenolate mofetil. When suspicion for psoriasis is strong providers should avoid systemic corticosteroids because of the risk of severe rebound of symptoms once off treatment. The initial approach can begin with attention to nutrition management, fluid and local skin care measures. Oatmeal baths as well as wet dressings can be used to cover affected areas followed by medium-potency topical corticosteroids [10]. Oral antihistamines can be used to relieve itching; systemic antibiotics may be used in those with infection. Patients with suspected drug hypersensitivity may be started on intravenous corticosteroid therapy. Biologic drugs or cyclosporine may be used in erythrodermic psoriasis [1]. Any suspected drug should be discontinued [10].

## Conclusions

Erythroderma is defined clinically as generalized erythema and scaling of the skin. It may be caused by multiple underlying etiologies, including atopic dermatitis, drug reactions, psoriasis, pityriasis rubra pilaris, cutaneous T-cell lymphoma and other less common causes. Many cases are “idiopathic” and an underlying etiology cannot be identified. Establishing the correct diagnosis involves correlation of the clinical and histopathologic findings. Treatment should be directed at the underlying dermatologic disease as well as towards managing systemic complications.
